# KRAS mutation in adenocarcinoma of the gastrointestinal type arising from a mature cystic teratoma of the ovary

**DOI:** 10.1186/s13048-014-0085-3

**Published:** 2014-09-06

**Authors:** Yan Li, Ruiguang Zhang, Danzhen Pan, Bangxing Huang, Mixia Weng, Xiu Nie

**Affiliations:** Department of Pathology, Union Hospital, Tongji Medical College, Huazhong University of Science and Technology, Wuhan, People’s Republic of China; Cancer Center, Union Hospital, Tongji Medical College, Huazhong University of Science and Technology, Wuhan, People’s Republic of China

**Keywords:** Ovary, Mature cystic teratoma, Malignant transformation, Adenocarcinoma, Intestinal type, KRAS mutation

## Abstract

Mature cystic teratomas (MCT) in the ovary rarely undergo malignant transformation. Moreover, adenocarcinoma of the gastrointestinal type is much rarer. We present two cases of perimenopausal female pateints with mature cystic teratoma of single ovary, while local adenocarcinoma arising in the MCT. The malignancies showed immunohistochemical features of intestinal differentiation, such as strong positivity for CDX-2, villin and CK-20, and negativity for CK-7. Furthermore, the mutation analysis of molecular alteration revealed a KRAS gene mutation in the intestinal adenocarcinoma part, extending into benign intestinal-type epithelium linings. Yet the mutation was not present in the epidermal component of the teratoma. We present these as two unique cases of mucinous adenocarcinoma of the intestinal type arising from mature cystic teratoma. Moreover, we also submit that this KRAS mutation might contribute to identify malignant transformation of a MCT and suggest possible effect on targeted treatment decisions for anti-epidermal growth factor receptor (EGFR) therapy in metastasized patients.

## Background

The most common tumor of ovary is mature cystic teratoma (MCT), which arises from germ cells and accounts for more than 20% of all ovarian tumors [1]. Malignant transformation of MCTs occurs in 1-2% of all these tumors [2]. During the malignancies arising in the MCT, squamous cell carcinoma is the most common type [3], with adenocarcinomas comprising only 6.8% of the malignant changes [4,5]. Furthermore, adenocarcinomas of the gastrointestinal type are much less frequent. So far there have been few reports of adenocarcinoma of the gastrointestinal type in the ovary arising from an MCT [6-8].

We present two cases with similarity of primary adenocarcinoma of the gastrointestinal type likely arising in a mature cystic teratoma of the ovary. Either patient had no evidence of a primary tumor elsewhere by previous medical history or radiographic survey. After extensive histological examination, the cyst with adenocarcinoma was found in continuity with a benign cystic lesion, which seemed at first slightly thicker compared to the surrounding normal wall. Yet, the microscopic evaluation revealed this part of cyst covered by epithelium showing malignant transformation into carcinoma with clearly defined irregular glands infiltrating into the stroma. The immunohistochemical (IHC) staining of the malignant change showed strong positivity for cytokeratin-20 (CK-20), villin and caudal type homeobox transcription factor 2 (CDX-2), while negative for CK-7. Moreover, as a number of studies had proved Kirsten rat sarcoma viral oncogene homolog (KRAS) and v-Raf murine sarcoma viral oncogene homolog B1 (BRAF) oncogene mutations are recognized biomarkers that predict lack of response to anti-epidermal growth factor receptor (EGFR) antibody therapies and are associated with a worse prognosis in patients with metastatic adenocarcinoma in colorectal cancer [9-11]. To investigate further treatment in case of recurrence and metastasis of intestinal carcinoma for those two patients, we evaluate molecular alteration on oncogene mutation in cysts carrying adenocarcinoma, which showed positive for KRAS mutation and the mutation was extended into the benign intestinal-type epithelium of the cyst.

Thus, we conclude that the adenocarcinoma of intestinal type was likely arising from a mature cystic teratoma. We also submit that this KRAS mutation might be helpful in identifying malignant transformation of a MCT and suggest possible effect on targeted treatment decisions for anti-epidermal growth factor receptor (EGFR) therapy in metastasized patients.

## Case presentation

The first patient was a 51-year-old perimenopausal female, gravida 3, para 1, who was admitted with occasional nausea, abdominal pain and bloating for the past few years. Her previous medical, surgical, gynecological, and family histories were all unremarkable, with regular menstrual cycle. A mass in the left adnexal region was found in a routine pelvic examination. Ultrasound scan revealed the presence of an approximately 6 cm complex cystic mass of the left ovary. And there were no abnormal findings of the uterus and right adnexae. The patient had slightly elevated serum level of only one tumor marker. Her carbohydrate atigen 19–9 (CA199) was elevated to 41.9 U/ml (normal < 35 U/ml), while the other serum tumor markers, including carcinoembrionic antigen (CEA), CA 125, α-fetoprotein(AFP), were all within the normal ranges.

An exploratory laparoscopy with frozen section of the left ovarian tumor was performed. After the result of frozen section confirming malignant change in MCT, the procedure was changed to a total hysterosalpingo-oophorectomy with 400 mg intraperitoneal carboplatin. Appendectomy and omentectomy were also carried out. Postoperatively, the patient opted for adjuvant chemotherapy, consisting of 6 cycles of paclitaxel (135 mg/m^2^) and carboplatin (area under the curve [AUC] of 5). After adjuvant chemotherapy, the patient’s CA199 went back to normal and a whole-body positron emission tomographic scan was performed, the result of which was negative. The patient remains free of disease 13 months following diagnosis.

Macroscopically, the left ovary contained a smooth cystic mass (5.8 cm × 4.5 cm) filling with sebaceous material, hair and teeth. The wall was average 0.1 cm - 0.3 cm thin. Most of the inner surface of the mass was smooth, and had partial thicking-like nodules lining a rough surface. The right ovary was 3.2 cm × 1.2 cm × 1.0 cm and otherwise unremarkable. The cervix contained 1 small mucous cyst, and the uterus contained 2 small leiomyomas (0.8 cm – 1.0 cm diameter).

**Figure 1:**
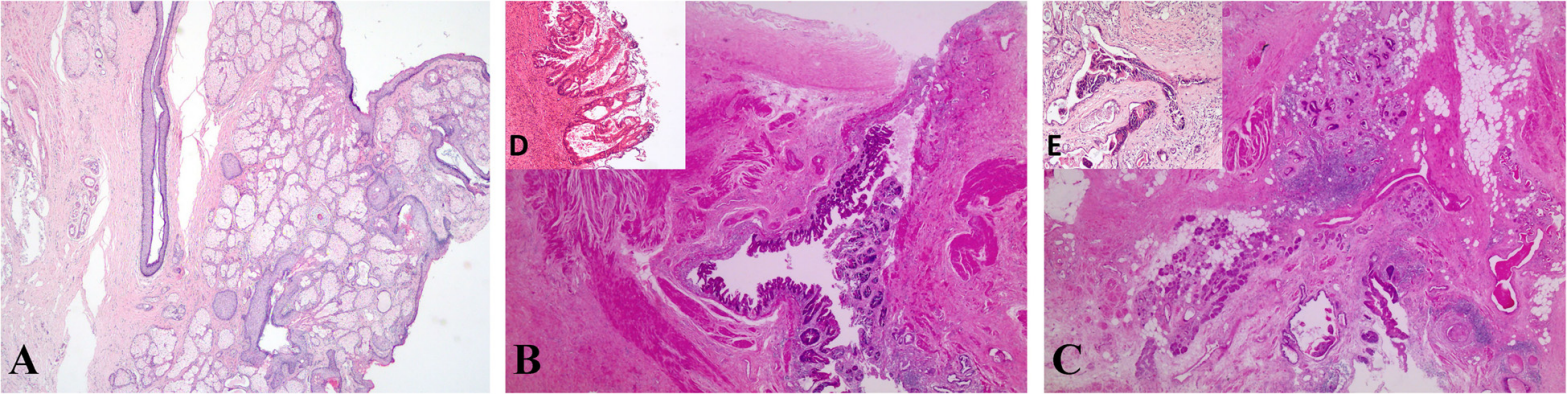
**The microscopic examination of the left ovarian mass. A**, Classic mature teratoma (haematoxylin - eosin, magnification x200); **B**, Malignant glandular epithelium was arising in continuity with benign mucous epithelium (haematoxylin - eosin, magnification x20); **C**, Tumour infiltration in the stroma (haematoxylin - eosin, magnification x20); **D**, Magnified malignant glandular epithelium of B from the same field (haematoxylin - eosin, magnification x 100); **E**, Magnified intestinal-type adenocarcinoma in the stroma of C from the same field (haematoxylin - eosin, magnification x 100).

**Figure 2:**
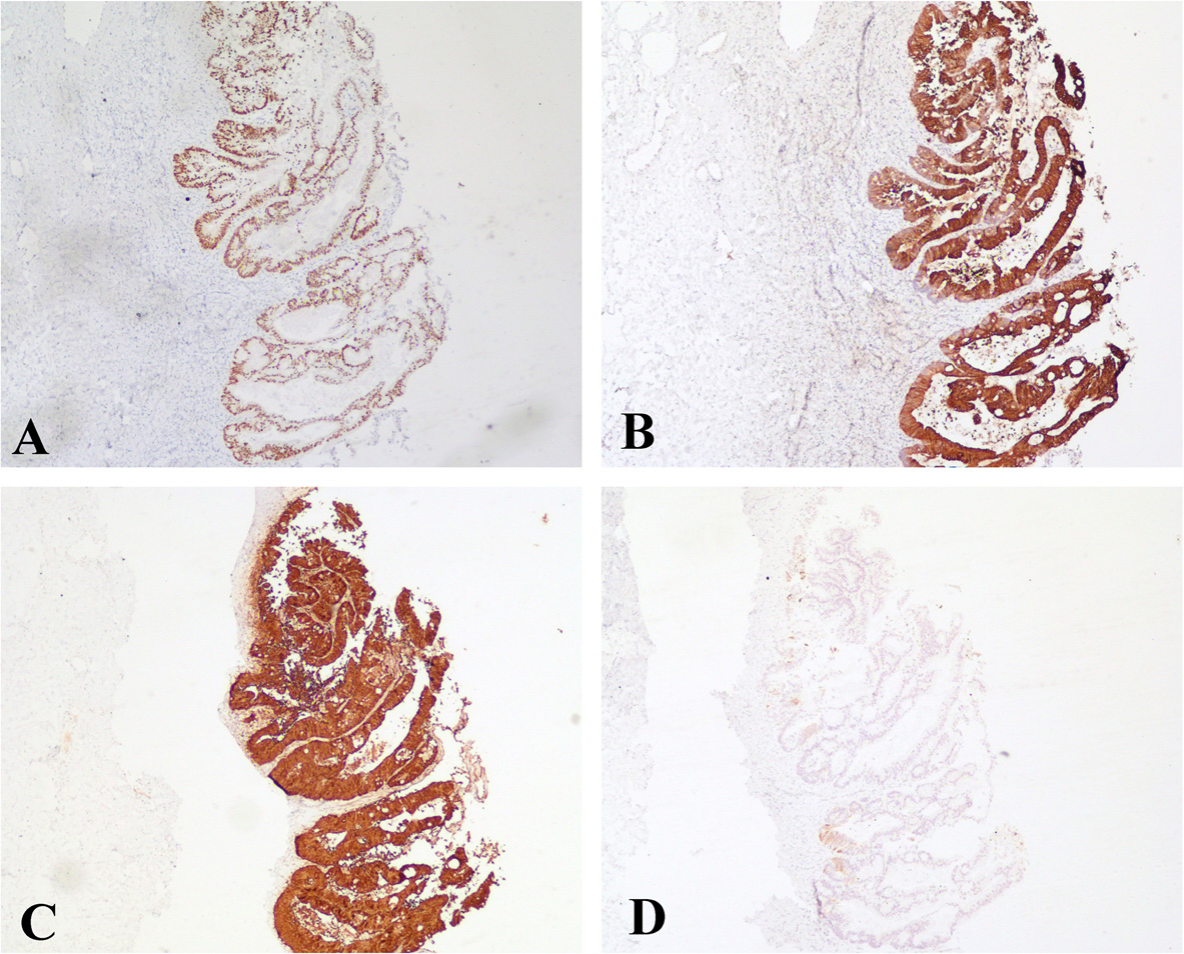
**Immunohistchemical staining of the adenocarcinoma part arising from an ovarian teratoma.** The adenocarcinoma part showed positive on CDX-2 **(A)**, CK-20 **(B)** and villin **(C)** while CK-7 **(D)** was negative (magnification x 400).

The microscopic examination of the left ovarian mass revealed components of a classic mature teratoma (Figure 1A). Most inner surface of the cyst was covered by squamous epithelium while malignant glandular epithelium (Figure 1B and D) was arising in continuity with benign mucous epithelium. The tumor was clearly defined irregular glands with stroma invasion (Figure 1C and E). Tumour cells showed IHC-positivity of CDX-2 (Figure 2A), CK-20 (Figure 2B), villin (Figure 2C). Moreover, the IHC analysis showed negativity of CK-7 (Figure 2D), oestrogen receptor (ER) and human epidermal growth factor 2 (HER2) (data not shown) in the adenocarcinoma part.

**Figure 3:**
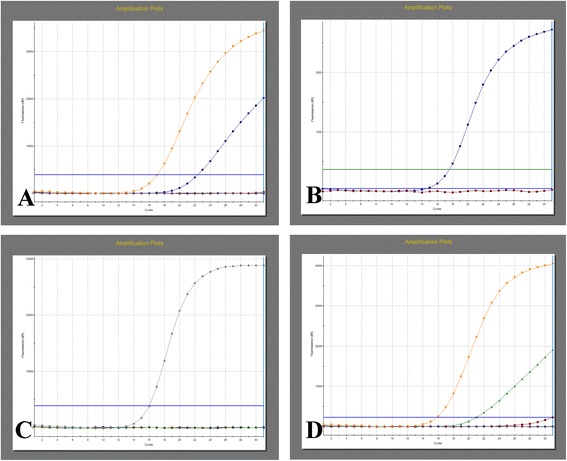
**Mutation analysis revealed a KRAS mutation not only in the adenocarcinoma part but also benign intestinal-type epithelium linings. A**, A mutation was found in codon 12 of the KRAS gene (NM_004985.3):c. 35G > T, p. (Gly12Val) in malignant glandular epithelium; **B** and **C**, There was no mutation in BRAF or EGFR genes in malignant glandular epithelium; **D**, The same mutation was found in benign intestinal-type epithelium in the same patient.

Genetic mutation was detected in various components of the tumor mass, including malignant adenocarcinoma, benign intestinal-type epithelium and the squamous epithelium of the mature cystic teratoma. EGFR inhibition-targeted therapy is a new biologic therapy for the treatment of metastatic colorectal cancer which is also adenocarcinoma of intestinal type [9,10]. KRAS was an oncogene whose mutation status is a prognostic factor for survival rate and predictive of response to EGFR inhibition-targeted therapy in intestinal adenocarcinoma [10-13]. BRAF [14,15] and EGFR (epidermal growth factor receptor) [16] genes, which are important co-factors of KRAS gene in the EGFR signaling pathway in the carcinogenesis, invasion and metastasis of colorectal cancers, also have prognostic value for patients with metastasis of adenocarcinoma. So we chose KRAS, BRAF and EGFR as targets of mutation detection. Interestingly, quantitative polymerase chain reaction (qPCR) revealed a mutation in codon 12 of the KRAS gene (NM_004985.3):c. 35G > T, p. (Gly12Val) in both the intestinal adenocarcinoma (Figure 3A) and benign intestinal-type epithelium (Figure 3D), yet no mutation was found in squamous epithelium of the mature cystic teratoma (data not shown). Furthermore, no mutations were found in BRAF (Figure 3B) or EGFR (Figure 3C) genes in either part.

The other patient was a 43-year-old perimenopausal female, gravida 2, para 1, presented in September 2012 for a routine pelvic examination; an adnexal mass was found. Her case was in highly similar to the first case but harboring a different KRAS mutation in codon 12 of the KRAS gene (NM_004985.3):c. 34G > T, p. (Gly12Cys). We summarized the relevant clinical and pathological findings of all the previous reports on adenocarcinoma of the gastrointestinal type arising from MCTs of the ovary and presented in Table 1.

**Table 1:** **Previous reports of adenocarcinoma of the gastrointestinal type arising from a MCT of the ovary**

**Case**	**Source**	**Age**	**Tumor marker**	**Tumor size (cm)**	**Surgical procedure**	**FIGO stage**	**Adjuvant therapy**	**Follow up**
1	Ueda G et al. [4]	62	N.E.	35	TAH + BSO	Ia	FAMT	15 years
2	Fishman A et al. [17]	38	CEA: 40 ng/ml CA 125: 80 U/ml CA153: 60 U/ml	20 × 13 × 8.5	TAH + BSO + OMT + APT	IIIc	5 FU Leucovorin	DOD 3 month after surgery
3	Kushima M [18]	52	CA19-9: 109 U/ml SLX: 58.5 U/ml CA125: 36 U/ml CA72-4: 19 U/ml	6.4 × 4.8 × 2.8	Bilateral SO	Ia	none	31 month
4	Levine DA [19]	37	CEA: 11.2 ng/ml CA 125, AFP, HCG: WNR	15 × 12 × 11	Unilateral SO + OMT + PLN + PAN	Ia	none	40 month
5	Guney M [20]	38	CA1 25: 99.1 U/ml CA19-9 > 1000 U/ml CEA: WNR	N.D.	TAH + BSO + OMT + PLN + PAN	Ia	none	N.D.
6	Min KJ [6]	77	CA125: 72 U/ml	17 × 14 × 2	TAH + BSO	Ia	none	12 month
7	Takai M [7]	49	CEA: 6.9 ng/ml CA 125: 20 U/ml CA19-9: 3.8 U/ml SCC: 1.1 ng/ml	6.7 × 5.7	TAH + BSO + OMT	Ic(b)	none	5 years
8	Dov Hershkovitz [21]	13	CA-19-9:162 U/ml CEA: 5.5 ng/ml CA-125: 268 U/ml AFP, HCG: WNR	7 × 10	N.D.	Ia	N.D.	5 month
9	Present case	51	CA19-9: 41.9 U/ml CEA, CA 125, AFP: WNR	5.8 × 4.5	TAH + SO + OMT + APT	Ia	P + C	13 month
		43	CA199 > 1200 U/ml	10.8 × 9.7	TAH + SO	Ia	none	11 month

1: TAH: total abdominal hysterectomy, SO: salpingo-oophorectomy, OMT: omentectomy, APT: Appendectomy, PLN: pelvic lymphadenecomy, PAN: para aortic lymphadenectomy.

2: FAMT: 5-furuorouracil + endoxan + mitomycin-C + toyomycin, P + C: paclitaxel + carboplatin.

3: N.E.: not examined, N.D.: no description, WNR: within normal ranges.

## Conclusions

We present two cases with similarity of primary adenocarcinoma of the gastrointestinal type, harboring KRAS oncogene mutation, likely arising in a mature cystic teratoma of the ovary. As far as we know, these two cases are unique because currently there have been few report on teratoma-associated adenocarcinoma carrying KRAS oncogene mutation [1,4-8,21,22].

Malignant transformation of MCTs is a rare, often asymptomatic event which most commonly occurs in postmenopausal women. Also there have been plenty of varying reports on that, the risk of malignancy is estimated to be between 0.17 – 2% among all the transformations. As expected, any component of the MCTs might undergo malignant transformation, but most of the time (about 80%) squamous epithelium is the most often prone to undergo malignant changes, which results in the squamous carcinoma [3]. Some less common malignancies include thyroid carcinomas [23], adenocarcinomas [1,4-7], or, less commonly, carcinoid tumors [24], mixed thyroid-carcinoid (struma carcinoid) [25].

The literature establishing adenocarcinoma of the gastrointestinal type is much rarer. To our knowledge, so far there have been only a few reports on it [4,6,7,17-19,21,22,26]. Although Kajo K *et al.* reported a case about mucinous carcinoma of non-intestinal type arising in the ovarian MCT [5], yet most of the previously described adenocarcinoma in MCTs showed characteristics of intestinal differentiation with immunohistochemical expression of CDX-2 and CK-20, and with negativity of CK-7 [7,22]. In present study, the immunohistochemical analysis showed positive staining in the CDX-2, CK-20 and villin, while the adenocarcinoma part was negative for CK-7, ER and HER2. The patient also underwent gastrointestinal endoscope to rule out the possibility of metastasis. Therefore, the diagnosis of present cases is adenocarcinoma of the intestinal type arising from a MCT.

The pathogenesis of mucinous adenocarcinoma arising from MCTs is somewhat different from that of colorectal cancer regarding clinical conditions. Morphology and immunophenotype of the intestinal type arising from a MCT are the same as in colorectal adenocarcinoma, more investigations are needed for further understanding pathogenesis particularly using molecular genetic analysis, like detection of mutation status. Although it has been claimed that colorectal adenocarcinoma is genetically similar to an intestinal-type adenocarcinoma in lung [20], so far there have been few reports on genetic comparison between colorectal adenocarcinoma and mucinous adenocarcinoma arising from MCTs. Reasons for the problem may be due to the limited case number and difficulty in separating the areas between the mucous components and adenocarcinoma part. Interestingly, recent studies on analysis of microsatellite polymorphism in mucinous ovarian carcinomas and associated teratomas showed a clonal match between the samples, suggesting that a subset of mucinous ovarian carcinoma actually arise from mature teratomas [27]. Fujii K et al. also demonstrated that the origin of the intestinal-type mucinous tumors might arise from mature cystic teratomas by microdissection and analysis of microsatellite markers [28]. Related studies gave us a clue to isolate different teratoma components and explore molecular genetic analysis.

It is difficult to make a diagnosis of malignant transformation of a MCT preoperatively. The patient’s age, extremely enlarged mass in adnexal region by imaging and elevated tumor maker might remind the possibility of malignancy. In preset study, both patients were perimenopausal. Not only were the imaging results abnormal, but also the tumor maker CA199 elevated. Yet, extensive histological sampling was still crucial to reveal the carcinoma part, especially for local malignancy. In present cases, mutation analysis of molecular alterations showed a KRAS mutation in both the adenocarcinoma part and benign intestinal-type epithelium of the cyst. KRAS mutations were observed in 43% of ovarian carcinomas and in 30% of colorectal carcinomas, which do not contribute to differential diagnosis in mucinous neoplasms of the colon and ovary [5]. Yet, mutation analysis might contribute to predict the malignant transformation in MCTs in case of under-diagnosis, especially for small lesion of malignancy. Moreover, the absence of KRAS mutation in the mature teratoma element in our samples suggests that this mutation accompanies with the intestinal-type epithelium.

The prognosis of patients with malignant transformation in MCTs is generally poor [29] and the majority of women die within a year after diagnosis [1]. The top affecting factor is tumor dissemination [30]. Thus, establishing an optimal therapeutic plan is important. So far, the treatment of choice for these tumors is surgery and there is little information on treatment outcomes of patients receiving chemotherapy directed to transformed histology given localized disease at presentation. Unfortunately, with tumors extending beyond the ovary, prognosis seems to be very poor. Dechaphunkul A *et al.* reported a case that a patient who achieved good response from chemotherapy directed to transformed histology, which confirms the importance of chemotherapy regimen used [31]. It brought us a new strategy of treatment by targeting at transformed histology. KRAS status is presently the only biomarker routinely used to select patients with colon cancer for EGFR inhibition-targeted therapy, which is widely used to treat metastatic colorectal cancer [32]. Thus we speculated if there was any chance for anti-EGFR antibody therapies after failure of first-line therapies, especially for metastasized patients. Further conclusions on selecting anti-EGFR antibody therapies need larger numbers of case studies and molecular genetic analysis in the intestinal-type adenocarcinoma arising from MCTs. Still, a risk and benefit discussion should be had with the patient, keeping in mind her age, comorbidities, surgical, and pathological findings.

Our case represents two unique cases of single ovarian MCT with malignant transformation to gastrolintestinal adenocarcinoma carrying KRAS mutation.

### Consent

Written informed consent was obtained from the patient for publication of this Case Report and any accompanying images. A copy of the written consent is available for review by the Editor-in-Chief of this journal.
